# Pandemic-Inspired Strategies for Cross-Sector Collaboration on Food Equity Across the Lifespan

**DOI:** 10.1093/geroni/igab046.2349

**Published:** 2021-12-17

**Authors:** Joan Ilardo, Angela Zell

**Affiliations:** Michigan State University College of Human Medicine, East Lansing, Michigan, United States

## Abstract

Early during the pandemic, access to food by residents across the lifespan was problematic in many communities. We observed well-intentioned responses by community organizations but a lack of centralized coordination across sectors, even as donations and resources significantly increased. Most of the organizations were in various sectors and not aware of the efforts and capabilities of others causing duplication or gaps in services. To prepare for future emergencies, our team created a project to develop and pilot a user-friendly, evidence-based roadmap to guide communities through the process of developing and sustaining effective collaborative partnerships for food and nutrition-related problems they could address together. We will describe the process through which we developed the roadmap structure and recruited stakeholders and content experts for our advisory board. To determine the effectiveness of our interventions, we designed methods with which we can analyze the organizations willing to use the roadmap and participate in the collaborative partnership; how they implement the roadmap; and ways they cope with challenges they face during implementation using strategies in the roadmap. We will describe elements of an effective, efficient roadmap development process using as many currently available evidence-based resources as possible and creating evidence-informed resources when we identify gaps. Expected outcomes are: 1) format of the final roadmap; 2) types of groups willing to use it; 3) how well the roadmap was implemented; 4) feasibility of continued use of the roadmap by groups over the long term; and 5) potential to expand roadmap use to other communities.

